# Prevalance of abnormal vault cytology after hysterectomy for cervical intraepithelial neoplasia, Pietermaritzburg

**DOI:** 10.4102/safp.v64i1.5457

**Published:** 2022-03-31

**Authors:** Sanele S. Mbuyisa, Thandekile L. Khumalo, Bongumusa S. Makhathini, Jagidesa Moodley

**Affiliations:** 1 Department of Obstetrics and Gynaecology, Faculty of Health Sciences, Grey’s Hospital, University of KwaZulu-Natal, Pietermaritzburg, South Africa; 2 Department of Obstetrics and Gynaecology, Faculty of Health Sciences, Edendale Regional Hospital, University of KwaZulu-Natal, Durban, South Africa; 3 Department of Obstetrics and Gynaecology, School of Clinical Medicine, University of KwaZulu-Natal, Durban, South Africa

**Keywords:** cervical intraepithelial neoplasia, recurrence rates, simple hysterectomy, vaginal intraepithelial neoplasia, vain

## Abstract

**Background:**

A simple hysterectomy is considered the definitive treatment modality for cervical intraepithelial neoplasia (CIN). However, it is associated with recurrence of vaginal intraepithelial neoplasia (VAIN) of up to 7.4%. We sought to determine recurrence rates of VAIN, in women living with HIV (WLWH) and non-infected women.

**Methods:**

This was a descriptive retrospective review of patients who received a simple hysterectomy for CIN between January 2015 and December 2017 in Pietermaritzburg.

**Results:**

Fifty-eight files were reviewed. Forty-two (72.4%) WLWH were seen; amongst those, 76.2% were virally suppressed. Abnormal vault cytology was only seen in patients with CIN grades 2 and 3. The recurrence rates for high-grade squamous intraepithelial lesion (HSIL) were 6.1% and 5.0% at 6 and 12 months, respectively. Human immunodeficiency virus co-infection was associated with 26.2% versus 13.3% of abnormal vault cytology compared to the HIV-negative counterparts (*p* = 0.164). Virologically suppressed patients had more abnormal cytology (28.1% vs. 0%) compared to the unsuppressed patients. There was a 17.2% and 65.5% loss- to follow-up rates at 6 and 12 months, respectively. Recurrence of premalignant lesions was noted amongst those who had the abdominal approach.

**Conclusion:**

The recurrence rates were comparable to the previous literature. A 6-month cytology follow-up showed no added benefit. Human immunodeficiency virus co-infection didn’t show a statistical significance on the recurrence rates; however, more structured studies are required to address this. Primary health care-based post operative surveillance can be a solution to address high loss to follow-up.

## Introduction

The general recurrence rate for cervical intraepithelial neoplasia (CIN) is between 1% and 21%, and 95% of these occur within the first five years after initial treatment.^1^ This recurrence is seen even in cases where clear pathologic margins are achieved with conisation, necessitating careful follow-up.^1^ Recurrent CIN lesions are, sometimes, treated with simple hysterectomy, and this is regarded as definitive management.^1,2^ Despite this approach, CIN and hysterectomy for CIN are known risk factors for vaginal intraepithelial neoplasia (VAIN) and recurrence rates of up to 7.4% have been reported.^1,2,3,4^ There is an increased risk (up to 60% – 70%) of vaginal cancer and VAIN in women with a history of CIN 3, and this remains increased after long-term follow-up of up to 20 years.^3^ There is evidence showing that women with a prior diagnosis of CIN 3, despite treatment, are at an increased risk of cervical or vaginal cancer that progresses with increasing age.^2,5^ This is thought to be because of the proximity of the vagina to the cervix, the loco-regional effect of human papilloma virus (HPV) and the woman’s inability to clear the infection.^3^

The risk is significantly lower in women with no history of CIN 3.^3^ There is no consistent evidence to support recommendations for maintaining ongoing cancer screening in women who have undergone hysterectomy with either HPV deoxyribonuclieic acid (DNA) testing or cytological evaluation.^6^ The South African guidelines for cervical cancer screening recommend that screening never ends for women living with HIV (WLWH) and should be scheduled at shorter intervals for women who have received treatment for premalignant disease.^7^

In the South African public sector, cervical cancer screening is conducted largely at primary care level institutions, which are mainly located within the community. Those with abnormal cervical cytology are treated at district hospitals or referred to the next level of care for further intervention. Primary care institutions are of utmost importance in quality implementation of cervical cancer primary and secondary prevention interventions, placing emphasis on the value of ongoing feedback between levels of care. The recommendation is that women who are not HIV infected exit screening at the age of 55 years or at hysterectomy.^7^ Cancer screening may be associated with anxiety for patients as false positive results may be seen and multiple consultations may be needed.^8^ Our study was conducted to determine the recurrence rate of vault premalignant lesions after a simple hysterectomy for premalignant cervical lesions as well as to determine the impact of the interval of performing vault cytology, with a special focus on WLWH.

## Materials and methods

**FIGURE 1:**
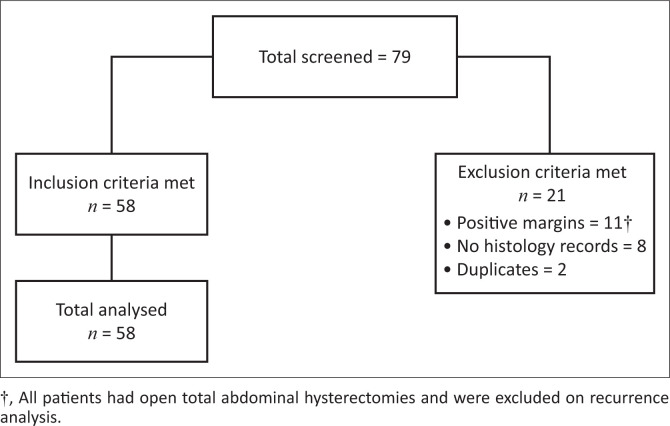
Flow diagram of the study population used for this study.

This was a retrospective study of women who had had a hysterectomy for CIN and follow-up cytology to determine recurrence rates, the impact of hysterectomy route and the impact of HIV co-infection on recurrence rates. Inclusion criteria were all adult women who had had a hysterectomy for premalignant lesions with a clear margin status on histology. Excluded were women who had had hysterectomies for other indications and where histology confirmed invasive cancer. Whilst those who had positive histological margins (11) were excluded from the recurrence analysis, they were included in the analysis of impact of the route. Figure 1 below demonstrates the study sample size.

### Statistical analysis

Graphical and descriptive statistics were carried out and bar graphs and tables were produced using Microsoft Statistical Package for the Social Science (SPSS) [AQ6]version 26. Parametric and non-parametric tests were used to determine associations between objectives and study variables. Statistically significant *p* = 0.05

### Ethical considerations

Ethical approval was provided by the Biomedical Research Ethics Committee of University of KwaZulu-Natal (BREC/00001269/2020, 09 June 2020). The study was registered with the National Health Research Database (NHRD) of South Africa (KZ_202005_009). The study was using patient records and no direct harm or risk was posed to human subjects.

## Results

A total of 79 files were retrieved within the study period from 01 January 2015 to 31 December 2017 in the Pietermaritzburg metropolitan area, under uMgungundlovu health district. Fifty-eight files met the inclusion criteria and were analysed. The mean age of the study group participants was 48.9 years with a range of 33 years to 76 years. Parity of the participants ranged between one and nine, with a mean parity of 3.64 children and a median of 3.0. Table 1 summarises the demographic details of the study sample.

**TABLE 1:** Demographic characteristics.

Characteristics	*n*	%
**Age categories (years)**		
25–35	1	1.7
36–45	22	37.9
46–55	23	39.7
> 55	12	20.7
Total	58	-
**Parity**		
P 0	0	0.0
P 1–4	43	74.1
≥ P5	15	25.9
Total	58	-
**Smoking status**		
Smoker	1	1.7
Non-smoker	18	31.03
Unknown	39	67.2
Total	58	-
**HIV status**		
Positive	42	72.4
Negative	15	25.9
Unknown	1	1.7
Total	58	-

HIV, human immunodeficiency virus.

Table 2 below summarises treatment details of women who were included in the study. A total of 68 prior interventions were performed on all 58 women as some women received more than one intervention.

**TABLE 2:** Treatment details of included women.

Finding	*n*	%
**Prior intervention (*n* = 68)**		
LLETZx1	47	69.1
LLETZx2	6	8.8
Cone biopsy	8	11.8
LLETZ + Cone biopsy	5	7.4
LLETZx2 + Cone biopsy	2	2.9
**Indication for hysterectomy**		
Persistent HSIL	43	74.1
CIN 3 + positive endocervical margins	6	10.3
Other	9	15.5
**Route of hysterectomy**		
Abdominal	52	89.7
Vaginal	6	10.3

LLETZ, large loop excision of the transformation zone; CIN 3, cervical intraepithelial neoplasia grade 3; HSIL, high grade squamous intraepithelial lesion.

The most commonly performed intervention prior to hysterectomy was a large loop excision of the transformation zone (LLETZ) at colposcopy with a frequency of 69.1% (*n* = 68). A total of five (8.8%) women received two interventions. The most common indication for hysterectomy was persistent high-grade squamous intraepithelial lesion (HSIL) in 43 (74.1%) women. Nine (15.5%) other indications (15.5%) were persistent low grade squamous intraepithelial lesion (LSIL) and women’s requests for hysterectomy (*n* = 4), and persistent atypical squamous cells – HSIL could not be excluded in five patients. Forty-two (72.4%) patients were living with HIV, 15 (25.9%) of them were non-infected and one patient’s status was unknown at the time of hysterectomy. Amongst the WLWH, 44 (76.2%) had a suppressed viral load, whilst 3 (4.7%) of them were on treatment but their viral load was not suppressed. The remaining 11 (19.0%) had unknown viral loads at time of surgery.

Open abdominal hysterectomy was performed more commonly than the vaginal route and a frequency of 89.7% was recorded compared to 10.3% of the patients who received vaginal hysterectomy. Table 3 below summarises cytology results of women who returned for follow-up at 6-month intervals. It also shows trends in the loss to follow-up rate over the course of time.

**TABLE 3:** Results of follow-up cytology.

Cytologic diagnosis	6-month follow-up (*n* = 49)		12-month follow-up (*n* = 20)		18-month follow-up (*n* = 9)	
	*n*	%	*n*	%	*n*	%
NILM	39	79.6	15	75.0	7	77.8
LSIL	2	4.0	1	5.0	1	11.1
ASC-US	1	2.0	1	5.0	0	0.0
ASC-H	1	2.0	0	0.0	0	0.0
HSIL	3	6.1	1	5.0	1	11.1
Deferred diagnosis	2	4.0	2	10.0	0	0.0
Lost to follow-up (*N* = 58 )	10	17.2	38	65.5	49	84.5

NILM, negative for intraepithelial lesion or malignancy; LSIL, low-grade intraepithelial lesion; ASC-US, atypical squamous cell of undetermined significance; ASC-H, atypical squamous cells-high grade intraepithelial lesion cannot be excluded; HSIL, high-grade squamous intraepithelial lesion.

The loss to follow-up rates was 10 (17.2%), 38 (65.5%) and 49 (84.5%) at 6 months, 12 months and 18 months, respectively. Post hysterectomy vault cytology with NILM results were 39 (79.6%), 15 (75.0%) and 7 (77.8%) at 6 months, 12 months and 18 months, respectively. The recurrence rates for HSIL ranged between 5.0% and 11.1% over a period of 18 months. Only three follow-up samples had no cytological diagnosis. No cytological diagnosis of VAIN was made at 6 months amongst those who had follow-up screening. In the HIV negative group, there was a 6.6% recurrence rate of abnormal cytology. One 50-year-old, HIV-negative patient presented with a vaginal lesion at 12 months follow-up and a punch biopsy confirmed vaginal cancer.

There was no statistically significant association between route of hysterectomy and recurrence rates (*p* = 1.000). Human immunodeficiency virus infection was associated with higher rates (26.2% vs. 13.3%) of abnormal cytology compared to HIV negative patients, but statistical significance could not be demonstrated (*p* = 0.164). Abnormal cytology was seen more frequently in virally suppressed patients (28.1% vs. 0%) than in those with unsuppressed viral loads, but this was not statistically significant (*p* = 0.425). Only patients diagnosed with CIN 2 and 3 at hysterectomy had abnormal cytology at 6 months, 12 months and 18 months follow-up. At 12 months, recurrence rates were 5.3% (*n* = 19) for both LSIL and HSIL (*p* = 0.511). Our data showed that all the patients who presented with abnormal vault cytology had been treated with abdominal hysterectomy. There were comparable rates of recurrence at 6 months and 12 months observed in our study.

## Discussion

The aim of the study was to evaluate the efficacy of a simple hysterectomy as a definitive intervention in the management of recurrent cervical premalignant lesions as well as to determine the effects of HIV co-infection on recurrence rates in this population.

### Time interval to positive cytology

The rates of abnormal vault cytology seen in our study were associated with the degree of dysplasia at the time of hysterectomy. Cervical intraepithelial neoplasia 2 and 3 were associated with higher rates of abnormal vault cytology results compared to medium risk premalignant lesions. This observation highlights the need for a more extensive risk-benefit counselling for patients with medium-risk premalignant lesions requesting a hysterectomy and ongoing follow-up compliance counselling to those with high grade lesions at hysterectomy. Cao et al. recommend a lifelong annual follow-up with cytology and HPV screening.^4^ The Royal Australian and New Zealand College of Obstetricians and Gynaecologists (RANZCOG) recommends annual cytology follow-up for a period of 5 years after hysterectomy.^9^ The observation of similar recurrence rates at 6 and 12 months in our study suggests that a 12 month follow-up strategy in line with the national guidelines is more cost effective.^7^ This recommendation incorporates and involves primary care level institutions that will carry out the ongoing screening. The majority of this study population was WLWH and had comparable recurrence rates to other studies (6.1%).^1,2,3,4^ Only one patient was HIV-negative, had CIN 3 and developed HSIL at the same time interval as the HIV-positive patients. Aberg et al. suggested that HIV infected women should receive cytological or HPV screening following hysterectomy, particularly if there was a history of CIN at or prior to surgery.^10^ Botha et al. recommend a lifelong annual surveillance of individuals who are co-infected with HIV in the South African population.^7^

### Route of hysterectomy on marginal clearance

The majority (89.7%) of this study population was treated with open simple abdominal hysterectomy in comparison to 10.3% patients who received a vaginal hysterectomy. Eleven patients, who were excluded from the study because of positive margins, all had open abdominal hysterectomies. Das et al. in 2005 found a margin positivity rate of 33.3% in the abdominal route compared to the vaginal route, which had only 3.3% positive margins.^11^ Based on their findings, they recommended that a vaginal approach be considered for premalignant cervical lesions because of the direct visualisation of the lesion at the time of procedure.^11^ One hundred percent of abnormal vault cytology was seen in abdominal hysterectomy in our study compared to 0.0% of those who had vaginal hysterectomy. Cheung et al. in China found recurrence rates of 25.0% for the vaginal hysterectomy subgroup compared to 40.0% from the abdominal hysterectomy group.^12^ Schockaert et al. in Belgium reported recurrence rates of 85.7% and 14.3% for abdominal and vaginal routes, respectively, for VAIN over time in their 2008 retrospective study.^1^ It seems pertinent to recommend a vaginal approach in patients with premalignant cervical lesions.

### Loss to follow-up

There was a high loss to follow-up rate in our study population. These findings are higher than the findings from a Belgian study which showed a loss to follow-up rate of 24.8% over 103 months at early intervals.^1^ This concerning finding could be because of multiple patient and healthcare system-related factors. A local study looking at follow-up of patients with gestational trophoblastic disease showed that socio-demographic factors, such as long distances travelled by patients, were associated with high loss to follow-up rates.^13^ This can potentially be mitigated by decentralising vault cytology follow-up to primary care level institutions, which deliver reproductive health services in the community. Although these findings were from a different study group with different follow-up interval protocols, it shares similar socio-demographic characteristics of a low income setting as our study population and the study was conducted in the same institution. These factors should ideally be addressed during pre-operative counselling and an individualised plan be tailored for those who cannot afford to comply with the recommended local protocol.^10^ Healthcare workers attending to patients treated for premalignant cervical lesions must be familiar with the recurrence rates so as to optimise the quality of follow-up and counselling, and primary care physicians need to be involved.

Short interval follow-up protocols could be another factor leading to high default rates, especially in low resource settings such as ours. Botha et al, similarly to Cao et al., recommend a yearly follow-up guideline for patients treated for pre-malignant lesions.^4,7^ Annual follow-up as well as the decentralisation of follow-up cytology to primary health care institutions could reduce the loss of follow-up demonstrated in our study by limiting number of visits to higher levels of care and alleviating the socio-economic burden to the population.

### Strengths and limitations

The findings from this study should be interpreted with caution because of its retrospective nature that makes it prone to bias limitations. The small sample size also compromises both clinical and statistical significance of the findings. However, it highlighted the clinical implications of the hysterectomy route for premalignant lesions and the probable socio-demographic challenges related to follow-up. These findings can be used as a platform for further research in the context of low resource settings. This study, in addition, has highlighted a gap in the system pertaining to down-referral patterns. Primary health care providers and tertiary institutions need to be made aware of this gap so that a collaborative strategy can be implemented, to improve referral frameworks geared towards patient retention.

## Conclusion

The recurrence rates for CIN were comparable to previous literature, particularly in women with a history of CIN grades 2 and 3. The risk is increased despite complete surgical excision. Clinicians need to acknowledge this risk and devise a careful follow-up strategy. Cytology at 6 months did not show added benefit. Human immunodeficiency virus co-infection did not show a statistical significance on the recurrence rates; however, better structured studies are required to address this issue further. High loss to follow-up rates needs a broader and multifactor oriented approach which should include a clear down-referral system to primary care institutions. Further studies are needed to investigate reasons for the high loss to follow-up rates in our population.
